# Clinic of Dr. Biddle

**Published:** 1856-03

**Authors:** 


*Clinic of Dr. Biddle.* Jan. 19, 1856. *Bright's Disease.*—Dr. Biddle presented to the class a case of *anasarca*, dependent on *albuminuria.* Dropsy, he observed, was not a disease in itself, but a symptom of disease; an effect rather than a cause. It was a morbid accumulation of the serum of the blood in the cellular tissue, or in some of the serous cavities of the body; and this might result from a variety of conditions. It might be produced by an obstruction in the circulation, preventing the return of the venous blood to the heart, as in valvular disease or dilatation of the heart. Or, the obstruction might exist in the liver, and the venous blood of the vena portæ, thrown back upon the capillaries of the abdominal viscera, would have its serum forced out into the peritoneal cavity, constituting ascites. A diseased condition of the serous membranes might be the cause of dropsical effusions into these cavities, as in chronic pleurisy and peritonitis. The state of the blood, too, might occasion effusion of its serum into the cellular tissue. Thus, the blood in anæmia, from a deficiency of its red-corpuscles, cannot always maintain sufficient vicidity to prevent a leakage of its water through the capillary vessels. And again, from the loss of another of its solid constituents, its albumen, the blood will become so thin that its serum will escape, and this was the explanation of the œdema in the case before them.

When the albumen of the blood escapes abnormally by the urinary excretion, as in the patient under notice, the term *albuminuria* is applied by modern pathologists to the morbid condition. But, the condition thus designated may depend upon very different pathological states of the kidneys; and it was most important, he observed, both for the prognosis and the treatment, correctly to distinguish the states of the kidneys upon which the albuminuria depended.

Since Dr. Bright first established the connection between albuminous urine and disease of the kidneys, the field of renal pathology thus opened had been assidiously cultivated; and it had been shown that there were two divisions of Bright's disease, differing most strikingly in the state of the kidneys with which they were associated, and also readily distinguishable during life. One of these varieties of Bright's disease was the result of a *desquamative disease* of the kidney; the other of *fatty degeneration.* It was sometimes erroneously asserted that the *desquamative* variety was always found in the *acute* form of the disease, as after scarlatina; and again, incorrectly, that the two abnormal states of the kidney were only successive stages of the same disease. Both the varieties of Bright's disease might, however, occur in the accute as well as chronic form; and they were essentially different in the character of the disease of the kidneys, as well as in the pathological appearance which these organs presented after death.

The great difference between the two forms of the disease, was in the morbid condition of the epithelial lining of the tubuli uriniferi of the kidneys. In one form, the epithelial cells underwent *desquamative inflammation;* they became disintegrated; were thrown off from the basement membrane, and were swept out from the tubules, entire, with the urine. The denuded tubules lost the power of secreting the ordinary solids of the urine, and, at the same time, were incapable of preventing the albumen of the blood from leaking through them with its water. In *fatty degeneration* of the kidney, the epithelial cells became the seat of the deposit of an excess of oil-globules; they underwent changes, varying in degree up to a complete oily degeneration; they were often distended by the oil-globules, and burst; but, although thus sometimes thrown off in fragments, they were not entirely detached, so as to leave the tubules bare. In the former condition, the denuded tubules allowed the transudation of a large amount of water in the urine; and hence a symptom of *desquamative* Bright's disease was a copious flow of urine, of light color, and low specific gravity. Not so, however, in *fatty degeneration;* here the condition of the tubules, while permitting the passage of albumen, was not favorable to the transudation of water, and a diminished amount of urine, of higher density, was the result. This was precisely the condition of the urine in the patient before them. The amount of urine passed was not more than two pints daily; its specific gravity was 1.020; and, as they saw, by the application of the tests, nitric acid and heat, the urine was loaded with albumen. By adverting to the probable condition of the kidneys in this case, they would understand why so large an amount of albumen was found in the urine. The kidney of *fatty degeneration* is increased in size; there is an excess—a true hypertrophy—of the glandular tissue; and the increased vascularity consequent thereupon was the cause of the abundance of albumen in the urine.

The character of the urine in the *desquamative* disease of the kidney is quite different. The amount secreted is copious, and its density low; its specific gravity is more often below than above 1.015 and sometimes as low as 1.005. The amount of albumen, too, found in the urine is much less; and this was easily understood by referring to the state of the kidney here. The kidney of desquamative inflammation was *small* and *contracted;* its vascularity diminished; its uriniferous tubes becoming more and more shrunken as the disease advanced; and as the supply of blood decreased, the materials for the secretion of albumen were also less.

The diagnosis, then, in this case, from the condition of the urine, was fatty degeneration of the kidney. The microscope would confirm this diagnosis; and must, indeed, always be resorted to, before they could pronounce with certainty as to the variety of renal disease upon which the albuminuria depended. But it would often be of great importance to them to be able to determine the nature of the affections upon mere inspection of the urine. In the case before them, the microscopical examination which would be applied would reveal the presence of a large amount of oil-globules in the sediment deposited by the urine with fragments of modified epithelial cells. In the urine of the small contracted kidney, on the other hand, are formed disintegrated epithelial cells, partly scattered, and partly in the form of tube-casts, but *few* or *no oil-globules.* A small amount of oil appears occasionally in the urine of desquamative disease, but never in any abundance, while the other appearances of the urine prevent any possibility of confounding such cases with fatty degeneration.

Dr. Biddle dwelt upon the importance of correctly distinguishing these two varieties of Bright's disease, because in the one form they might expect much from treatment, while in such cases as that before them, recovery was nearly hopeless. Dr. Wilks, of Guy's Hospital, London, goes so far as to assign three years as the maximum of time which a patient, attacked with fatty degeneration of the kidney, has to live. This was, perhaps, too exclusive; but, in the present case, the prognosis was rendered still more unfavorable by a complication of hypertrophy of the heart. Of the many complications which coexisted with Bright's disease, this was the most common, as well as the most alarming. About one-half the cases presented this form of heart disease, and more than two-thirds of them were accompanied by some variety of cardiac affection.

Dr. Biddle called attention to the insidious establishment of the affection in this case. There was nothing to invite attention to the kidneys as a seat of disease. There had never been pain in the loins, and, indeed, there very seldom was in chronic cases. The treatment here could be only palliative. The age of the patient (sixty-five), removed almost absolutely the hope of a return of the kidneys to the normal condition. And, so far as they had to do with remedial agents, they were entirely at a loss for any class which had a known efficacy in restoring glandular tissue which had undergone fatty degeneration. Still, something might be done to abate the dropsical effusion, and to support the nutrition of the patient. He should prescribe ten grains of nitrate of potassa, and a grain of digitalis, three times a day, with a view to an increased action in the eliminating water of the kidneys, and, at the same time, to a sedative influence on the heart. The constant drain upon the albumen of the blood which was going on, demanded the freest use of strong animal food. A little wine would also be proper.

In the very forming stage of fatty disease of the kidney, an alterative course, which would induce a powerful modifying influence on the nutritive functions, might do something to prevent the advance of this degeneration. Hence the importance of determining the condition, in its very commencement, and, with this view, of an early examination of the urine, in all cases of doubtful chronic disorder. Before the recurrence of albuminuria, the microscope will often show the existence of the disease. And it should also be borne in mind that *heat*, carefully applied, will readily detect a slight amount of albumen, which would be decomposed by nitric acid. To distinguish albumen coagulated by heat, from a phosphatic deposit, a few drops of acetic acid should be added, which will redissolve a phosphatic sediment.—*Med. and Surg. Reporter, N. Y.*

